# Preliminary study on dosimetry characteristics of a novel cylindrical dose verification system

**DOI:** 10.1002/acm2.14138

**Published:** 2023-09-04

**Authors:** Long He, Jinhan Zhu, Xuetao Wang, Bailin Zhang, Qiang Hu, Lixin Chen, Xiaowei Liu

**Affiliations:** ^1^ School of Physics Sun Yat‐sen University Guangzhou China; ^2^ Sun Yat‐sen University Cancer Center Guangzhou China; ^3^ Radiation Oncology Department The Second Affiliated Hospital of Guangzhou University of Chinese Medicine Guangzhou China; ^4^ Guangzhou Raydose Medical Technology Company Limited Guangzhou China

**Keywords:** detector array, dose verification, dosimetry, ionization chamber

## Abstract

**Objective:**

To develop a novel ionization chamber array dosimetry system, study its dosimetry characteristics, and perform preliminary tests for plan dose verification.

**Methods:**

The dosimetry characteristics of this new array were tested, including short‐term and long‐term reproducibility, dose linearity, dose rate dependence, field size dependence, and angular dependence. The open field and MLC field plans were designed for dose testing. Randomly select 30 patient treatment plans (10 intensity‐modulated radiation therapy [IMRT] plans and 20 volumetric modulated arc therapy [VMAT] plans) that have undergone dose verification using Portal Dosimetry to perform verification measurement and evaluate dose verification test results.

**Results:**

The dosimetry characteristics of the arrays all performed well. The gamma passing rates (3%/2 mm) were more than 96% for the combined open field and MLC field plans. The average gamma pass rates were (99.54 ± 0.58)% and (96.70 ± 3.41)% for the 10 IMRT plans and (99.32 ± 0.89)% and (94.91 ± 6.01)% for the 20 VMAT plans at the 3%/2 mm and 2%/2 mm criteria, respectively, which is similar to the Portal Dosimetry's measurement results.

**Conclusions:**

This novel ionization chamber array demonstrates good dosimetry characteristics and is suitable for clinical IMRT and VMAT plan verifications.

## 1 INTRODUCTION

Recently, the use of complex treatment delivery techniques in radiotherapy, such as intensity‐modulated radiation therapy (IMRT) and volumetric modulated arc therapy (VMAT), has become increasingly widespread in clinical tumor treatments.^1^, ^2^, ^3^ However, their complexity in planning design and delivery requires a standard and strict quality assurance (QA) procedure to guarantee patients’ safety and treatment delivery accuracy. As an important quality control method, plan dose verification is necessary for the QA process.^4^, ^5^, ^6^, ^7^

Three common methods can be used to perform IMRT dose verification: (1) the true composite (TC) delivery method uses the actual treatment plan parameters for the patient to perform measurements to verify the composite dose distribution; (2) the perpendicular field‐by‐field (PFF) measurement compares the planned versus measured dose for each perpendicular field; and (3) the perpendicular composite (PC) measurement obtains a single dose image integrated for all the perpendicular fields for analysis. For pretreatment dose verification, the TC delivery method provides the closest simulation of the treatment delivered to the patient.^8^

Several well‐established commercial devices are available for pretreatment dose verification, including diode arrays, ionization chamber arrays, etc.^9^, ^10^, ^11^, ^12^, ^13^ These devices can be used to perform QA measurements using suitable methods based on their properties. For example, planar ionization chamber detector arrays may be suitable for measurements perpendicular to the direction of beam incidence. If used for TC dose verification measurements, the measurement phantom must be of sufficient thickness when the gantry is rotated to the horizontal or near‐horizontal horizontal position of the detector array, otherwise, the effective measurement area of the array for lateral beams appears to be insufficient. The cylindrical phantom keeps the effective measurement area constant during gantry rotation, and typical representatives include the diode arrays Delta4(Scandidos AB, Uppsala Sweden) and ArcCHECK (Sun Nuclear Corp, USA), both of which use cylindrical phantom that can effectively avoid these defects. In addition, planar array detectors need to be corrected for angular dependence relative to the beam incidence direction, otherwise, the measurement accuracy in composite dose verification is compromised.^14^, ^15^, ^16^, ^17^

To improve the inherent drawbacks of planar ionization chamber arrays and inspired by the cylindrical phantom design, a novel cylindrical array system (ArcMap, RayDose, China) was designed for pretreatment dose verification, to be used for TC dose verification measurements with rotational delivery techniques. On the one hand, the dosimetry characteristics of this new ionization chamber array were evaluated, and in addition, preliminary tests of clinical treatment plan dose verification were performed to evaluate the applicability of this array for IMRT and VMAT verifications. This study is expected to provide basic reference data for more clinical application studies of this system in the future.

## 2 MATERIAL AND METHODS

### 2.1 Dosimetry system

The designed novel dose verification system consists of an ionization chamber array, and software for dose analysis. The array had 1764 cylindrical air ionization chamber detectors embedded inside the cylindrical phantom, forming 21 large detector rings (21 cm in diameter) and 21 small detector rings (19 cm in diameter). The rings were arranged in parallel and alternately along the axis of the phantom. By adopting this arrangement pattern, 1764 ion chambers formed two cylindrical measurement surfaces with different depths inside the phantom. Each measurement surface consisted of 21 rings of the same diameter spaced at 1.02 cm. Each ring had 42 evenly distributed ionization chambers with an angular spacing of 8.57°. The cylindrical measurement surfaces with diameters of 21 and 19 cm are denoted as “outer‐arc” and “inner‐arc,” respectively. An offset of 0.5 cm exists between the outer‐arc and inner‐arc along the phantom's axis. The chamber diameter of the ionization chamber is 6 mm, the chamber height is 4.8 mm, and the chamber volume is 0.135 cm^3^.

**FIGURE 1:**
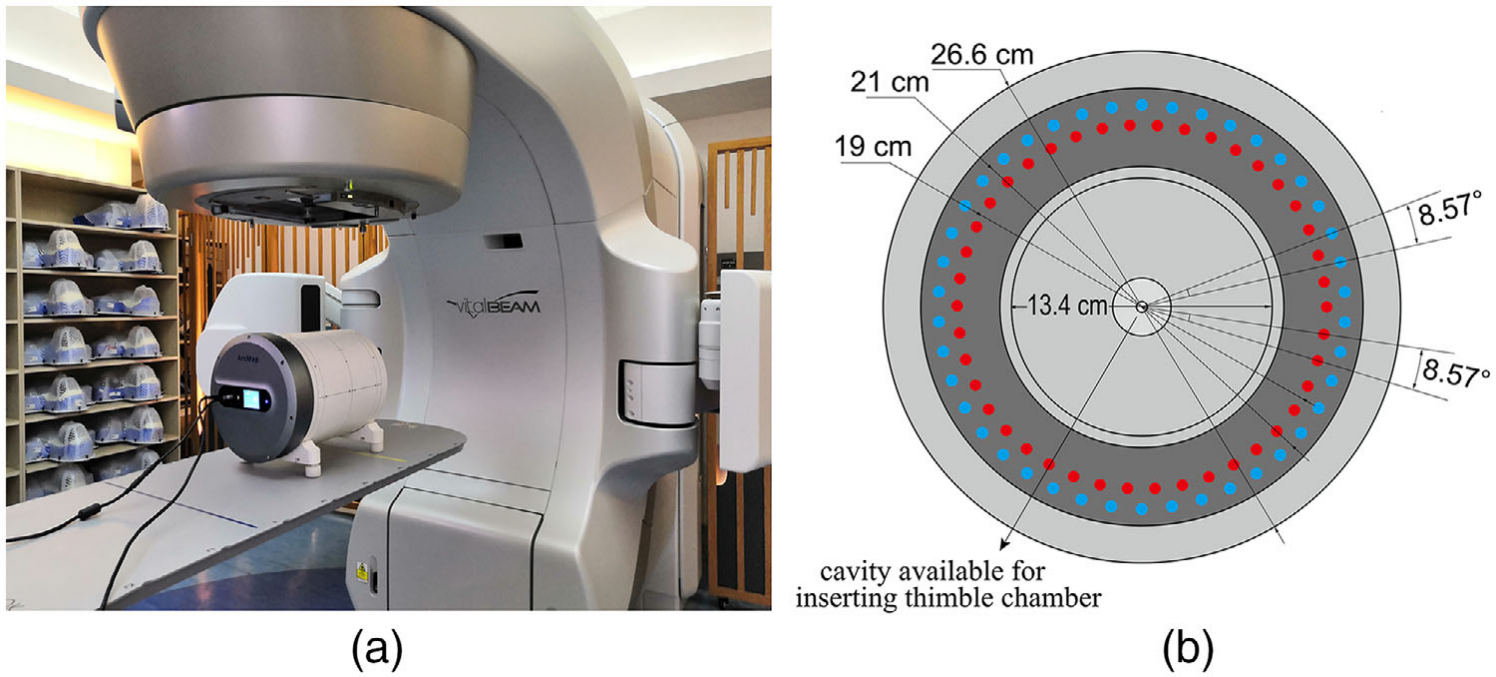
ArcMap dose verification system: (a) dosimetry device; (b) axial cross‐section of phantom and internal detector geometry sketch diagram: the inner and outer detector rings have an axial offset; the homogeneous plug with a diameter of 13.4 cm: the central cavity can accommodate a thimble chamber.

The length of the measurement phantom is 29.5 cm and the physical density of the material is 1.04 to 1.06 g/cm^3^. The physical area of the measurement (detector array) is 21 × 21 cm^2^. The measurement phantom was a hollow cylinder (13.4 cm and 26.6 cm inner and outer diameters, respectively), on the one hand, to reduce the weight of the equipment and to facilitate its use; it is also used to accommodate plugs made of homogeneous or non‐homogeneous materials with a diameter of 13.4 cm, and a cavity in the center of plug is designed to accommodate a thimble chamber for measuring the absolute dose of the cylindrical phantom center. The software system for dose analysis displays in real time the dose images collected on both measurement surfaces of the array; and compares and analyzes the measured dose distribution with the reference dose distribution. Figure 1 illustrates the ArcMap dose verification system and its internal structure.

### 2.2 Linear accelerator and treatment planning system

The research work was mainly performed with a 6 MV x‐ray beam from a linear accelerator (Vitalbeam, Varian, USA). The accelerator was equipped with 60 pairs of MLC leaves, with a maximum field size of 40 × 40 cm^2^. The plans were generated in the Varian's Eclipse 15.5 Treatment Planning System (TPS) using the anisotropic analytical algorithm (AAA, version 15.5.12) and a 2 mm grid size for dose calculation.

### 2.3 Dosimetry characteristics measurement

In this study, the phantom with a homogeneous plug was used to perform measurements. The cylindrical phantom's axis was aligned with the accelerator gantry's rotation axis and the phantom's geometric center coincided with the accelerator's isocenter in all the measurements unless specified otherwise. A 0.6 cc thimble chamber (PTW30013) was inserted into the ArcMap phantom to measure the output of the accelerator output at the isocenter as a reference.

#### 2.3.1 Array calibration

In order to correct the sensitivity bias caused by the difference between detectors of the ionization chamber array, the array needs to be calibrated first. Since the detectors are interleaved in the inner‐arc and outer‐arc, they are different from the single plane distribution. On the basis of referring to the principle of existing methods, an autonomous optimization of the calibration method was carried out.^18^ The ionization chambers on the top of the central ring of the outer‐arc and inner‐arc are selected as a calibration reference for the rest of the detectors on the two measurement surfaces, respectively. ArcMap array calibration was performed with 20 radiation exposures at 10 different setup requirements, identified as steps A through J (each step was repeated twice). For each step, the accelerator is set to a nominal dose of 100 MU. The phantom is in the SAD setup, which is the initial position for the array calibration. The array calibration procedure consists of the following four parts:

1. Part I: With the phantom in the initial position, set the field size to 28 × 30 cm^2^, rotate the gantry to 0°, and then irradiate the array with a nominal dose of 100 MU. Then, rotate the gantry to 17° and 60° to perform the same operation (corresponding to steps A, B, and C).
2. Part II: Rotate the gantry to 0° and set the field size to 28 × 30 cm^2^. From the initial calibration position, move the couch 10.2 mm toward the G direction of the accelerator gun‐target direction (let the ionization chamber longitudinally adjacent to the reference chamber be positioned in the center of the beam), and then irradiate the array. Next, move the couch 10.2 mm from the initial calibration position toward the T direction and then irradiate the array (corresponding to steps D and E).
3. Part III: Similar to Part II above, but the couch moving distance needs to be adjusted to 96.9 mm (let the ionization chamber at the edge of the array be positioned in the center of the beam), and the field size is set to 7 × 5 cm^2^ (corresponding to steps F and G).
4. Part IV: Similar to Part I above, but the phantom must be rotated 180° along the axis, and the remaining operations remain unchanged (corresponding to steps H, I, and J).

After the calibration procedure was completed, the calibration result of the detector array was verified by two parts of measurements: (1) Angle rotation measurement: the accelerator delivered 100 MU beam each time around the phantom at a gantry interval of 30°, with a field size is 25 × 25 cm^2^; the phantom was rotated 180° around its axis when irradiating the bottom half. After the measurements were completed, the array acquired post‐irradiation dose images of beams incident from 12 different directions (12 images from each of the two measurement surfaces). (2) Axial movement measurement: with a field size of 25 × 10 cm^2^ and the gantry position of 0°, three different areas of the detector array were exposed to the irradiation field in turn by moving the treatment couch, and the accelerator delivered 100 MU beams each time.

#### 2.3.2 Reproducibility

Measurements were made with a 10 × 10 cm^2^ field size and 400 MU/min dose rate. At a gantry of 0°, the accelerator delivered 100 MU each time. The short‐term reproducibility was evaluated by calculating the maximum deviation (MD) and standard deviation (SD) of 10 consecutive measurement readings of the ionization chambers in the central region of the irradiation. In addition, the measurement was taken once a month and repeated six times to evaluate the long‐term reproducibility. The ArcMap software was used to record the corresponding measurement results. During the measurement, the thimble chamber was placed at the isocenter to obtain the absolute dose to correct the variation in the accelerator output.

#### 2.3.3 Dose linearity

Measurements were performed with a 10 × 10 cm^2^ field size and repeated thrice. The accelerator delivered beam doses of 1−600 MU at a 0° gantry position. The measured results were averaged to eliminate uncertainty in the measurement. The readings of the ionization chambers in the central region of the field were selected for dose linearity analysis. For each measurement, the thimble chamber was used to measure the absolute dose of the isocenter to correct the variation in the accelerator output.

#### 2.3.4 Dose rate dependence

Measurements were performed with a field size of 10 × 10 cm^2^. The accelerator delivered 100 MU beams each time at regular dose rates of 20, 40, 80, 100, 200, 300, 400, 500, and 600 MU/min in 6X energy, respectively. In the 6X‐FFF energy, 100 MU was delivered each time at a high dose rate of 600, 800, 1000, 1200, and 1400 MU/min, respectively, and the array then performed dose measurements at a sampling rate of 50 and 100 ms, respectively. The dose rate dependence of the ionization chamber was evaluated by measuring the responses of the ArcMap array. A thimble chamber was used to correct the variation of the accelerator output. The identical measurements were repeated thrice, and the results were averaged.

#### 2.3.5 Field size dependence

Field size dependence of the ionization chambers was evaluated using 100 MU delivery for six field sizes (3 × 3 cm^2^, 5 × 5 cm^2^, 7 × 7 cm^2^, 10 × 10 cm^2^, 15 × 15 cm^2^, and 20 × 20 cm^2^) at a gantry of 0°. The measurements were repeated thrice at the same field size, and the results were averaged. The measured readings of the detectors at the center of the field were selected as the target values and compared with the TPS‐calculated values.

#### 2.3.6 Angular dependence

To measure the directional responses of the array detectors inside phantom, the selected ion chamber was positioned at the accelerator isocenter. Measurements were performed with a 10 × 10 cm^2^ field size, on a varying gantry angle. The gantry was rotated counterclockwise from 105° to 255°, with 100 MU beam output per 15°. To avoid couch attenuation when irradiation the bottom half, the phantom was rotated 180° about its axis and the selected detector was repositioned at the isocenter; the gantry was rotated counterclockwise from 90° to 270°, with 100 MU of beam output per 15°. Three identical measurements were repeated to reduce the uncertainty during the measurement process. Furthermore, two different ionization chambers were selected to evaluate their directional responses difference. The TPS‐calculated doses corresponding to each measurement condition were obtained as reference to evaluate the angular dependence of the array.

#### 2.3.7 Combined open field testing

Two testing cases of combined open field were designed in this study: (1) a combined field plan consisting of 3, 5, 10, 15, and 20 cm square fields with a gantry of 0° and 100 MU delivery each field; (2) one four‐field box irradiation (box filed) plan consisting of four 20 × 20 cm^2^ fields, with gantry angles of 0°, 90°, 180°, and 270°. Compare the measured dose distributions by ArcMap with the calculated dose distributions by TPS.

#### 2.3.8 MLC field testing

Shaper software (Varian Corporation, USA) was used to design two types of MLC field plans. Static MLC field: MLCs employ a step‐and‐shoot method within a 20 × 16 cm^2^ field, with the leaf position on the Bank B side kept fixed and the leaf on the Bank A side moved sequentially with a 4‐cm spacing to form five subfields. Dynamic MLC field: MLCs employ a sliding window method in a 24 × 20 cm^2^ field to move with a 4‐cm subfield width. At the gantry of 0°, each MLC field plan was performed once at 0° and once at 90° of the collimator, respectively, with 100 MU delivery per subfield. Two testing cases described above were measured using the ArcMap system and compared with the TPS‐calculated dose distribution.

### 2.4 Patient‐specific IMRT and VMAT plan dose verification

Twenty VMAT plans and ten IMRT plans were randomly selected from the patients' clinical treatment plans, and these plans were ported to the computed tomography (CT) images of the ArcMap phantom to create the corresponding verification plans. Among them, all the IMRT plans employed a dynamic method; all the VMAT plans consisted of two full treatment arcs. The measurements of the verification plans were performed using the actual gantry positions of the treatment plans. The difference between the measured TC dose distribution and the calculated dose distribution was compared.

### 2.5 Evaluation method

The gamma analysis method was used to compare the measured dose distribution with the TPS‐calculated dose distribution. Both surfaces of measurements were combined to perform the gamma analysis and calculate global gamma passing rates (GPRs). The measured results were used as a reference distribution, and the TPS‐calculated dose distribution was linearly interpolated according to a 1 mm grid and then compared with the measured dose distribution. Different dose difference tolerances and distance‐to‐agreement (DTA) tolerances were chosen as criteria to compute gamma indices. In this study, the ArcMap‐measured dose distribution and the TPS‐calculated dose distribution were compared using three different criteria (3%/3, 3%/2, and 2%/2 mm). Gamma analysis was performed in the absolute dose mode, with a low‐dose threshold of 10%.

## 3 RESULTS

### 3.1 Array calibration

**FIGURE 2:**
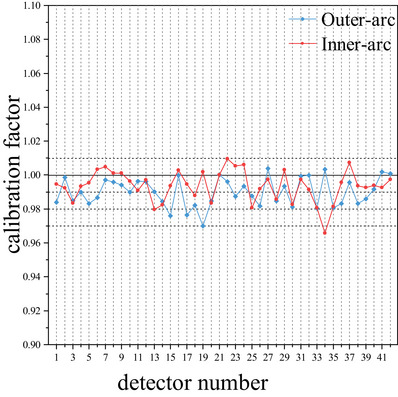
The calibration factors of 42 chambers on the center ring of outer‐arc and inner‐arc.

**FIGURE 3:**
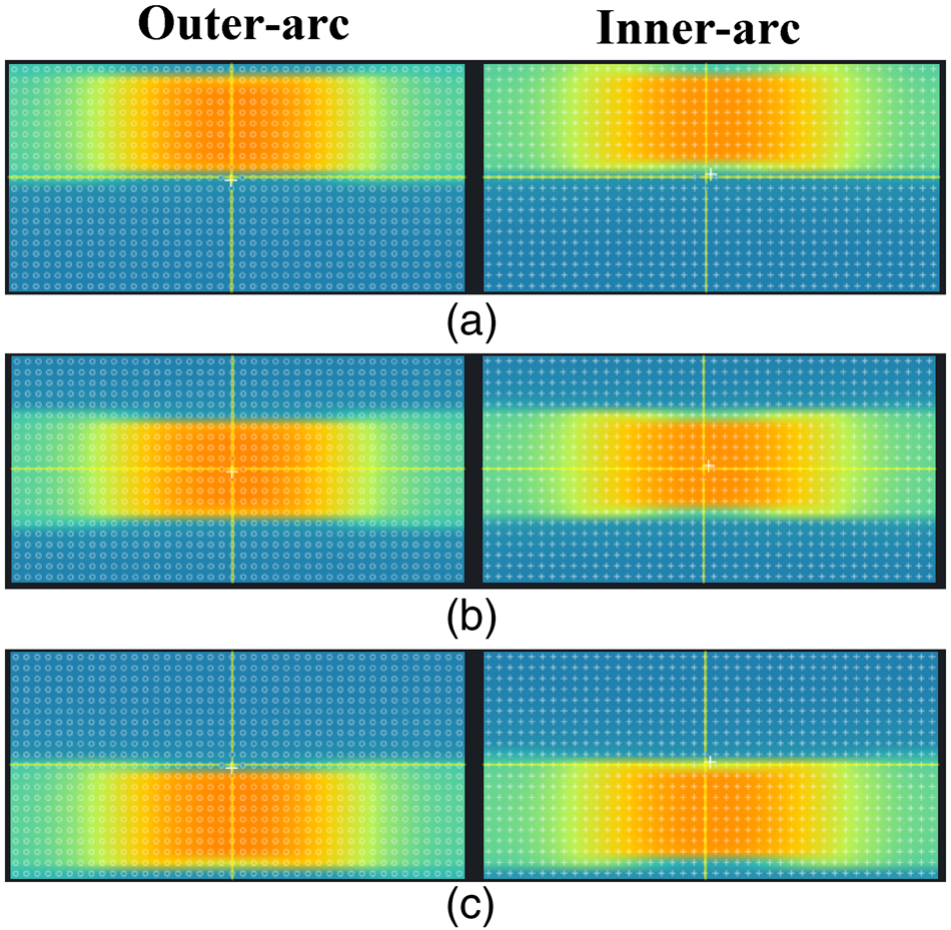
Dose images acquired by the array with the ArcMap phantom moved along the axial direction under a field of 25 × 10 cm^2^. If the array was cut from its bottom, the ionization chambers of ArcMap will be spread on two planes. The positions of the dose pictures acquired in each of the three areas of the array are shown schematically in Figures (a), (b), and (c), respectively.

The calibration factors of the inner‐arc and outer‐arc detectors range from 0.9657 to 1.0395 and 0.9609 to 1.0473, respectively. Figure 2 shows the calibration factors of 42 chambers on the center ring of outer‐arc and inner‐arc. Due to the cylindrical geometry of the array, we can consider the two measurement surfaces of the array as consisting of 42 generatrix parallel to the axis of the cylindrical phantom, and number each generatrix from 0° to 360° in clockwise order, starting from the generatrix on the top of the array (right above) to correspond to the angle position of the gantry during rotation. In the angle rotation measurements, the array measurements under gantry of 0° and 30° were used as two reference dose distributions, and were circularly shifted and aligned with the remaining measured images, respectively. Then the dose distributions between them were compared. Table 1 summarizes the gamma analysis results between dose distributions measured by the array. In the axial movement measurement, three different areas of the array were irradiated and three dose images were acquired (Figure 3). The measured dose distributions were compared to each other, the total GPRs were 98.47% (579 out of 588 dose points), 99.83% (587 out of 588 dose points), and 97.79% (575 out of 588 dose points) at the 1%/1 mm criterion, respectively. Both parts of the verification measurements demonstrated high passing rates, indicating good calibration results for the array.

**TABLE 1:** Gamma analysis results between measured dose distributions.

Reference	Passing rate	Outer‐arc		Inner‐arc	
		γ (1%/1 mm)	γ (2%/1 mm)	γ (1%/1 mm)	γ (2%/1 mm)
^a^Map0°	Average	95.01%	100%	98.59%	99.98%
	Std Dev	0.028	0.001	0.010	0
Map30°	Average	93.74%	100%	98.55%	100%
	Std Dev	0.023	0.005	0.006	0

^a^ Map0° represents the dose distribution measured by the ArcMap array where the y‐axis of the irradiation field coincides with the 0°‐generatrix of the array.

### 3.2 Reproducibility

**FIGURE 4:**
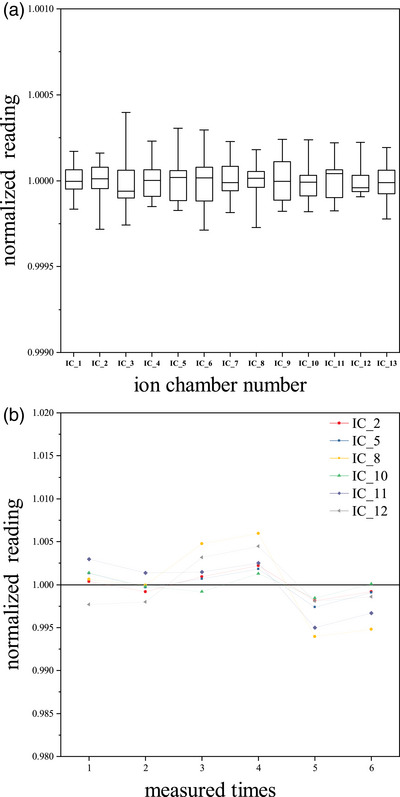
Array reproducibility measurement results. (a) Short‐term reproducibility of 13 ionization chambers at the central region of the array. (b) Long‐term reproducibility of 6 ionization chambers.

Thirteen ionization chambers closest to the central axis of the field were selected to evaluate the reproducibility. Nine ionization chambers were in outer‐arc and numbered from 1 to 9, and four ionization chambers were in inner‐arc and numbered from 10 to 13. IC_5 refers to the top central ionization chamber (IC) at the outer‐arc. Figure 4a shows the short‐term reproducibility of the 13 ionization chamber detectors. The measurement results of each ion chamber were normalized to the average of 10 consecutive measurements. The SDs of 13 ion chambers for 10 consecutive measurements were less than 0.02%, and the MDs were less than 0.04%. Figure 4b shows the long‐term reproducibility, for all selected detectors, the SD was less than 0.41%, and the MD was less than 0.61%.

### 3.3 Dose linearity

**FIGURE 5:**
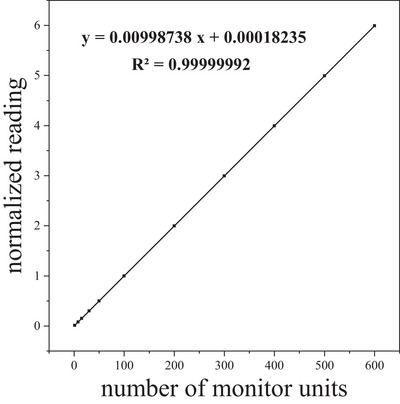
Dose linearity measurement results. The normalized measured readings in the figure came from the detector IC_5.

The measured readings of the ionization chambers in the central region of the beam were normalized and linearly fitted with accelerator output. The selected detectors showed good dose linearity, and the *R* ^2^ value of the fitted lines was better than 0.99999. Figure 5 shows the detector's dose linearity compared with the accelerator output. The discrepancy between the actual measured value and the theoretical values of the corresponding fitting line was within 0.2%.

### 3.4 Dose rate dependence

**FIGURE 6:**
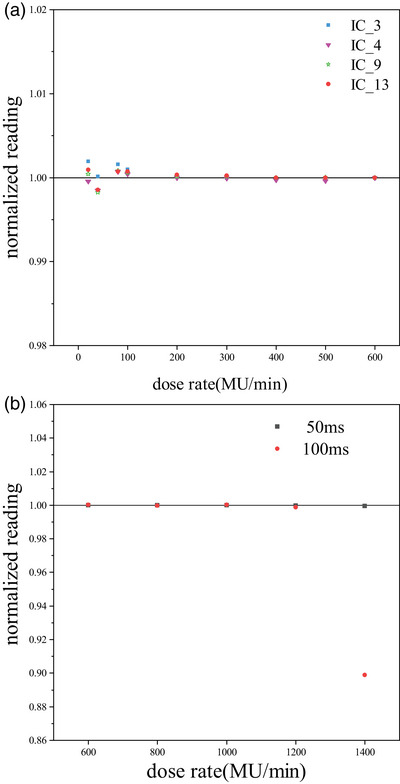
Dose rate response of the ArcMap array. (a) 6 MV mode, measurements of the ionization chamber at regular dose rate (only 4 are shown) and (b) 6 MV‐FFF mode, measurements of the IC_5 at high and low sampling rates at high dose rates.

Figure 6a shows the dose rate dependence of the array between 20 and 600 MU/min. Each measured reading of all the selected ionization chambers was normalized to their respective measurements at the 600 MU/min. From the normalized measurements, the response variation was less than 0.2% and the SD was less than 0.08% for all selected ionization chambers at different dose rates. Figure 6b shows the measurements of the IC_5 at both high and low sampling rates in the dose rate range of 600 to 1400 MU/min. Each measured reading was normalized to the measurements of the 600 MU/min at sampling rates of 50 ms. The response variation at different dose rates at 50 ms is less than 0.05% and the SD is less than 0.02%. However, when the measurements were performed at a sampling rate of 100 ms, the variation of the ionization chamber response was less than 0.15% over the dose rate range of 600 to 1200 MU/min, and at a dose rate of 1400 MU/min, the response of the ionization chamber decreased by 10.12%.

### 3.5 Field size dependence

**FIGURE 7:**
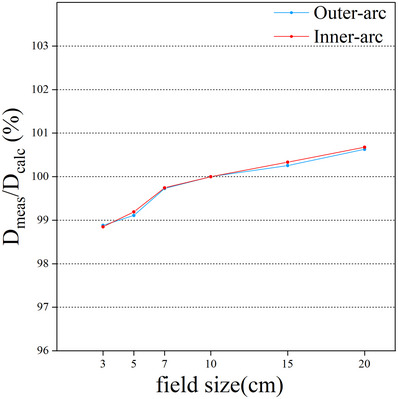
The field size dependence of ArcMap ionization chamber for field sizes of 3 × 3 cm^2^ to 20 × 20 cm^2^. D_meas_/D_calc_ indicates the ratio of ArcMap measured results to TPS calculated results.

As the irradiation field increased, the responses of the array increased. The readings of the selected ionization chambers were normalized with respect to their respective readings for the 10 × 10 cm^2^ field. Figure 7 shows the field size dependence of the two ionization chambers located on different measurement surfaces. Comparing the ArcMap measurements with the TPS calculations, the discrepancies are about −1.1% for the field of 3 cm by 3 cm, and the discrepancies are all within ± 1% for the remaining fields.

### 3.6 Angular dependence

**FIGURE 8:**
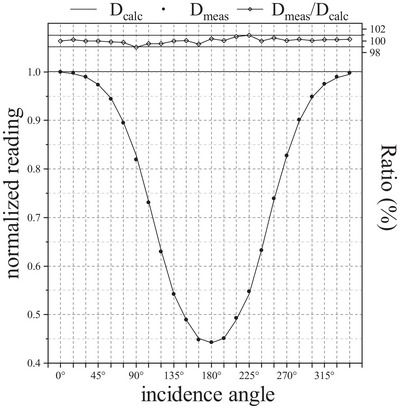
Angular dependence of the ArcMap array detector. D_meas_/D_calc_ indicates the ratio of ArcMap measurement to TPS calculation.

Figure 8 shows the chamber angular dependence determined by comparing relative chamber response to TPS‐calculated reference. The measured values of the detector and the calculated values of the TPS were normalized to their respective value at the beam incidence of 0°. Compared with the TPS‐calculated reference value, the difference between the measured and calculated results of the ionization chamber is essentially within ± 1%; the maximum discrepancy is 1.08% when the angle of incidence is 90°. Directional responses of two selected ion chambers normalized to their respective response at the normal beam incidence exhibited similar pattern, their difference at 105° is 0.83%, which is the only result greater than 0.7%, and the differences are less than 0.4% for 15 of the 24 angles.

### 3.7 Combined open field testing

**FIGURE 9:**
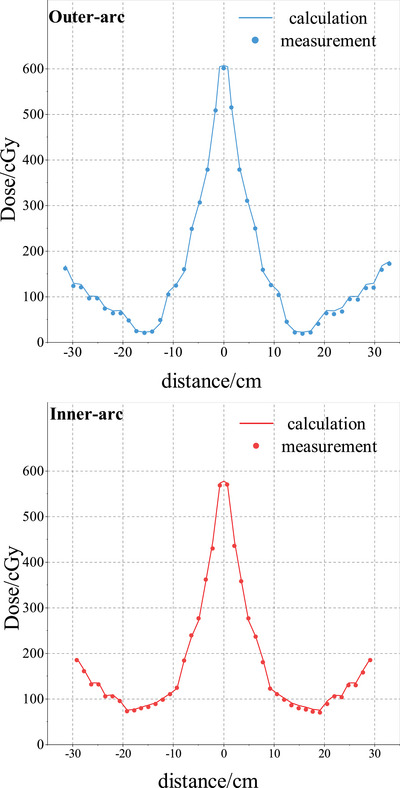
Comparison of the dose profiles from TPS calculation and array measurement for combined field plan. The abscissa indicates arc distance along detectors ring.

The measured dose distributions of the two open field plans were compared with the reference dose distributions calculated by TPS at the 3%/2 mm criterion. The number of dose points passes and the corresponding GPRs for the array are given in Table 2. The passing rates for two plans are better than 96%. Figure 9 shows the dose profiles for the combined field plan.

**TABLE 2:** The results of gamma analysis of the dose distribution for the combined open field plans and MLC field plans at 3%/2 mm criteria.

Number of points with γ ≤ 1	Outer‐arc		Inner‐arc		Total passing (%)
	Pass	Fail	Pass	Fail	
Combined field	465	1	715	0	99.92
Box field	868	14	834	48	96.49
Static MLC	475	28	589	16	96.02
Dynamic MLC	827	10	832	9	98.87

### 3.8 MLC field testing

**FIGURE 10:**
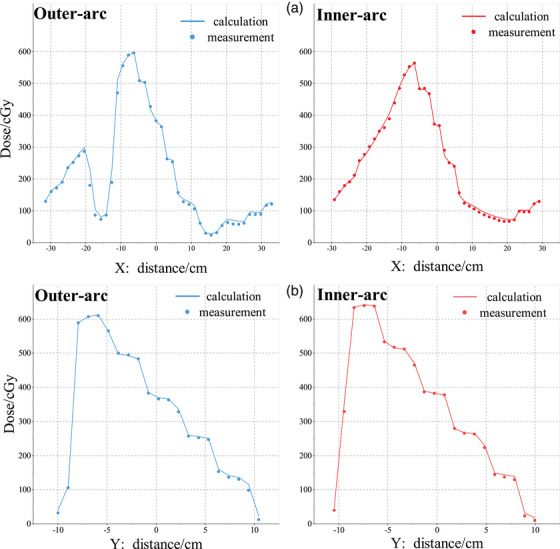
Comparison of the dose profiles from TPS calculation and array measurement for static MLC plans with (a) collimator of 0° and 90° collimator of 90°. The abscissa indicates arc distance along detectors ring.

**FIGURE 11:**
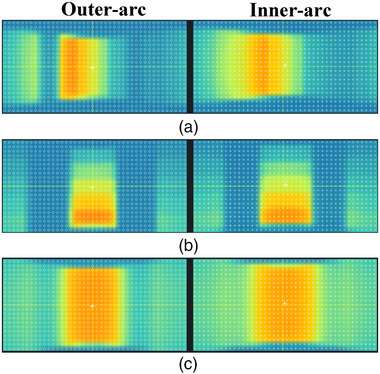
Dose images acquired by two measuring surfaces of the array. (a) static MLC filed plan with collimators of 0°, (b) static MLC filed plan with collimator of 90°, (c) dynamic MLC field plan with collimator of 0°.

The test results of the MLC field are given in Table 2. The passing rates for all plans are better than 96% at 3%/2 mm criteria. Figure 10 shows the dose profiles for the static MLC field plans with collimators of 0° and 90°, respectively. Figure 11 displays the dose images corresponding to the two MLC field plans acquired at each of the two measurement surfaces.

### 3.9 Patient‐specific IMRT and VMAT plans dose verification

The results of the gamma analysis for dose verification measurements of all patient plans are shown in Table 3. At the 3%/2 mm criterion, the average GPRs were better than 98% and 96% for all IMRT and VMAT plans, respectively. At the 2%/2 mm criterion, the average GPRs were 96.70% and 94.91% for the 10 IMRT plans and 20 VMAT plans, respectively.

**TABLE 3:** The gamma analysis results of IMRT and VMAT plans.

Passing rate of γ (%)	IMRT			VMAT		
	Mean ± SD	Max	Min	Mean ± SD	Max	Min
3%/3 mm	99.75 ± 0.34	100	98.90	99.86 ± 0.225	100	98.97
3%/2 mm	99.54 ± 0.58	100	98.02	99.32 ± 0.87	100	96.46
2%/2 mm	96.70 ± 3.41	99.64	87.62	94.91 ± 6.01	100	79.73

Abbreviations: IMRT, intensity‐modulated radiation therapy; VMAT, volumetric modulated arc therapy.

## 4 DISCUSSION

In the dose verification measurements of IMRT and VMAT, the detector used for dosimetry should be guaranteed in terms of accuracy, including detector linearity, reproducibility, and the response of the detector to the ray incidence angle, which will affect the measurement results. In designing and studying this novel ionization chamber array measurement system, the following aspects were considered: (1) the ionization chamber was selected as the detector of this system, mainly because the ionization chamber is one of the most classic detectors, and the ionization chamber also has its own inherent advantages compared with other detectors, such as good long‐term stability and sensitivity independent of the accumulated irradiation dose; (2) the ionization chamber is arranged in an axial manner, which can cleverly avoid the influence of different incidence angles of radiation on the response of the ionization chamber in the range of 360°; (3) the detectors are arranged on two cylindrical surfaces in stagger manner, which can effectively increase the spatial resolution and avoid that the ray path under certain angles (such as near 90° or 270°) passes through more cavities before reaching the detector.

Whether the tool is suitable for practical verification measurement, it needs to be fully tested first. This includes array calibration and dosimetry characteristics study. This study first tested the accuracy of the array calibration method, and the results showed that the inner and outer ionization chambers can meet the requirements of clinical application after calibration. In addition, periodic array calibration will also help identify the detector units that may have problems in time and improve the accuracy of the measured dose.

Detector reproducibility and dose linearity are two important indicators for clinical applications. From the above measurements, the dose verification system also shows good measurement reproducibility (short‐term and long‐term) and dose linearity. The array has no dose rate dependence over the tested dose rate range and can be applied to variable dose rate radiotherapy techniques, such as VMAT.

Several different methods have been used to evaluate the angular dependence of the ArcCHECK array, and this study refers to the measurement method used by Li et al.^9^, ^19^, ^20^ Placing the selected ionization chamber in the accelerator's isocenter can accomplish that the detector is always located in the center of the irradiation field, which can make the measurements and TPS calculations more accurate. In addition, we performed a test when the array was isocenter setup: the responses of the detector with clockwise gantry rotation from 340° to 20° were measured with an interval of 2° (corresponding to an incidence angle range of −22.3° to 22.3°). The measured values varied between −0.24% and 0.35% compared to the corresponding TPS‐calculated values. The measurements show that the ArcMap array has a low angular dependence, which is due to the use of cylindrical ionization chambers in the ArcMap, and each detector axis is parallel to the axis of the phantom. In this respect, the ionization chamber has some advantages over the diode detectors.^21^ However, for noncoplanar arcs, which are not covered in this work, further studies are needed.

Before dose verification, open field testing can usually be used to determine the current working state of the machine, such as the deviation of the output, and also to find out more easily if there are problems with the measuring tool. Two testing cases were designed in this work, a combined field formed by different size square fields under 0° irradiation, and a box irradiation field under different angle irradiation. On the one hand, the steepness of the gradient of the single field penumbra region was appropriately reduced to avoid the obvious influence of the setup deviation on the test results, and on the other hand, it also takes into account the reason that the phantom is cylindrical rather than flat. The test results showed good results, but it should be noted that when using box irradiation, the attenuation effect of the couch needs to be taken into account. Therefore, three‐field irradiation can also be considered as a testing case (excluding 180° incidence).

Significant deviations in the MLC leaf position accuracy during IMRT and VMAT plans delivery is one of the most important factors affecting the accuracy of dose delivery and is one of the more likely problems to occur. Therefore, testing the MLC field before verification, or in case of poor dose results are found, can help to solve the related problems. In this work, static and dynamic MLC field testing cases were designed, and the tool was tried to test the MLC field after the MLC leaves were maintained and serviced. The test results are good and can be used as a benchmark for detecting MLC status at work. How to use this tool to test the MLC leaf position accuracy and what kind of accuracy can be achieved will need to be continued in the next research.^22^

Compared to a single plane of a two‐dimensional detector array, the dual‐layer design of the ArcMap array in three‐dimensional space improves spatial resolution. It is worth noting that the effective measurement areas of the two measurement surfaces are different, and the corresponding detector densities also differ. The ArcMap array can directly obtain some dosimetry information at two different depths for analysis while helping to better reconstruct the three‐dimensional dose. Based on the current dual‐layer design, the diameter of the inner measurement surface can be considered to be further reduced, which would enable the inner measurement surface to be closer to the high‐dose region during plans verification measurement.

In this study, 30 randomly selected clinical plans have completed dose verification using Portal Dosimetry (Varian) before patient treatment. The EPID has high resolution and ensures that the detection plane is always perpendicular to the beams during the measurement. The average GPR of verification results using the Portal Dosimetry was 98.91% ± 1.53 at the 3%/2 mm criterion. 30 plans were verified using the ArcMap system, and the GPRs of all plans were better than 96% at the 3%/2 mm criterion. A paired t‐test revealed no significant discrepancy between the GPRs evaluated by the ArcMap and the GPRs evaluated by the Portal Dosimetry, with a corresponding *p*‐value of 0.46 (>0.05). Plans for the nasopharyngeal sits are usually more complex than those for other sites, and randomly selected plans included 13 nasopharyngeal VMAT plans (all consisting of 2 full treatment arcs). Due to the good dosimetry characteristics of the ArcMap array (no dose rate dependence, low angular response dependence, etc.), it was found that the gamma analysis results still had high passing rates when using the system to verify complex VMAT plans.

## 5 CONCLUSIONS

We have designed a new ionization chamber array. It is shown that this dose verification system has good performance in dosimetry and can meet the requirements of detectors for clinical applications. Preliminary tests on clinical plans have demonstrated the applicability and effectiveness of dose verification for VMAT and IMRT plans while providing a basic reference for further studies on the clinical application of the tool. This tool can further enrich the choice of dose verification tools for physicists.

## AUTHOR CONTRIBUTIONS

Long He contributed to the project design, made all measurements, performed the data analysis, and wrote the manuscript. Jinhan Zhu contributed to the project design, measurements, and data analysis. Xuetao Wang, Bailin Zhang, and Qiang Hu participated in measurements and data analysis. Lixin Chen and Xiaowei Liu initiated this project, contributed to its design, and edited the manuscript. Xiaowei Liu oversaw the general progress, and determined the final version of the manuscript.

## CONFLICT OF INTEREST STATEMENT

The authors declare no conflict of interest related to this work.

## Data Availability

The data that support the findings of this study are available from the corresponding author upon reasonable request.
